# Neuralgia of the Uterus Arising from Dentition

**Published:** 1846-10

**Authors:** R. Davis

**Affiliations:** London


*Neuralgia of the Uterus arising from Dentition.* By R. Davis, Esq., M. R. C. S., London.—Mrs. S., of Shoreditch, a lady of full habit, and twenty-five years of age, was delivered of a male child on the 3rd of September, 1844. The labour was one of some difficulty, it being a breech presentation, and it was, after the expulsion of the child, attended with considerable hæmorrhage. It is curious here to observe, that out of the five children of which this lady has been delivered, four were breech presentations. There was adhesion of the placenta, which required the introduction of the hand into the womb to effect its removal. With the exception of some fainting, and a few after-pains, everything went on favourably until the sixth day after delivery, when she was attacked with pain of a severe character in the region of the uterus. There was no fever, no heat of skin, no derangement of the circulating or digestive systems, and no increase of pain upon pressing the abdomen. For the relief of this pain, opiates and other sedatives were freely administered, both in a solid and liquid form, without benefit. We may here observe, that although the pain was of a most severe character, it was intermittent in its attacks, and during the intermissions pain was referred to the face, but not considered of such consequence by the patient as to inform me of the circumstance. The pain of the abdomen continuing, and fearing that this irritation might terminate in inflammation, leeches, fomentations, mustard plasters, bran poultices, and blisters were applied freely, and a pill, consisting of one grain of opium, and two of calomel, was administered every three hours. The pain in the face now became more severe, and the patient, supposing it to arise from toothache, surrounded the parts with flannel. This circumstance led to inquiries on my part, which ended in an examination of of the mouth, when I found the posterior part of the gum enlarged, red, and swollen. The mystery was explained. One of the dentes sapientiæ, the cause of all the pain in the uterus, was coming up; the gum was freely lanced, and the pain in the uterus from that moment subsided. No more medicine was required or given.—*London Lancet.*

